# Impact of Vitamins, Antibiotics, Probiotics, and History of COVID-19 on the Gut Microbiome in Ulcerative Colitis Patients: A Cross-Sectional Study

**DOI:** 10.3390/medicina61020284

**Published:** 2025-02-07

**Authors:** Zane Straume, Nikola Krūmiņa, Ilze Elbere, Maija Rozenberga, Renārs Erts, Dace Rudzīte, Anna Proskurina, Angelika Krumina

**Affiliations:** 1 Gastroenterology, Hepatology and Nutrition Clinic, Riga East Clinical University Hospital, LV-1038 Riga, Latvia; dace.rudzite@aslimnica.lv; 2 Department of Internal Diseases, Riga Stradins University, LV-1007 Riga, Latvia; kruminanikola@gmail.com (N.K.); anna.proskr@gmail.com (A.P.); angelika.krumina@rsu.lv (A.K.); 3 Latvian Biomedical Research and Study Centre, LV-1067 Riga, Latvia; ilze.elbere@biomed.lu.lv (I.E.); maija.rozenberga@biomed.lu.lv (M.R.); 4 The Faculty of Medicine and Life Sciences, University of Latvia, LV-1004 Riga, Latvia; renars.erts@lu.lv; 5 Pauls Stradins Clinical University Hospital, LV-1002 Riga, Latvia

**Keywords:** ulcerative colitis, microbiome, probiotics, vitamins, antibiotics, COVID-19

## Abstract

*Background and Objectives*: The human gut microbiome is essential for the health of the host and is affected by antibiotics and coronavirus disease 2019 (COVID-19). The gut microbiome is recognized as a contributing factor in the development of ulcerative colitis. Specific vitamins and probiotics have been demonstrated to positively influence the microbiome by enhancing the prevalence of expected beneficial microorganisms. *Materials and Methods*: Forty-nine ulcerative colitis (UC) outpatients from Riga East Clinical University Hospital were enrolled in this cross-sectional study from June 2021 to December 2021. All patients were divided into groups based on history of COVID-19 (COVID-19 positive vs. COVID-19 negative) in the last six months. Information about antibiotic, probiotic, and vitamin intake were outlined, and faecal samples were collected. The MetaPhlAn v.2.6.0 tool was used for the taxonomic classification of the gut microbiome metagenome data. Statistical analysis was performed using R 4.2.1. *Results*: Of the 49 patients enrolled, 31 (63%) were male and 18 (37%) were female. Coronavirus disease 2019 was found in 14 (28.6%) patients in the last 6 months. *Verrucomicrobia* was statistically significantly lower in the COVID-19 positive group (M = 0.05; SD = 0.11) compared to the COVID-19 negative group (M = 0.5; SD = 1.22), *p* = 0.03. Antibiotic non-users had more *Firmicutes* in their microbiome than antibiotic users (*p* = 0.008). The most used vitamin supplement was vitamin D (N = 18), fifteen (42.9%) of the patients were COVID-19 negative and 3 (21.4%) were COVID-19 positive over the last six months (*p* > 0.05). Vitamin C users had more *Firmicutes* in their gut microbiome compared to non-users (Md = 72.8 [IQR: 66.6; 78.7] vs. Md = 60.1 [IQR: 42.4; 67.7]), *p* = 0.01. *Conclusions*: Antibiotic non-users had more *Firmicutes* than antibiotic users in their gut microbiome. Only vitamin C had statistically significant results; in users, more *Firmicutes* were observed. A mild course of COVID-19 may not influence ulcerative colitis patients’ gut microbiome.

## 1. Introduction

The human microbiota contains ten times more bacterial cells than human tissue cells, and there are a hundred times more bacterial genes compared to human genes [1,2,3]. These bacteria are found on all surfaces of the human body, including the gastrointestinal and respiratory systems. The microbiota serves various roles, including the digestion and absorption of nutrients, the production of vitamins, and the stimulation, guidance, and regulation of host immunity. Maintaining gut microbiota balance and its potential antiviral properties has garnered significant attention. Dysbiosis, an imbalance in gut microbiota, has been observed in individuals with various gastrointestinal diseases and plays a significant role in the development of ulcerative colitis. Different factors like antibiotics and COVID-19 can affect gut microbiota [4,5,6,7,8].

Ulcerative colitis is characterized by inflammation of the colon, which can lead to dysbiosis in the gut microbiota, potentially exacerbating the condition. The pathogenesis of ulcerative colitis involves a complex immune response against the intestinal microbiota, influenced by factors such as genetics, environmental factors, and lifestyle choices. The complete molecular mechanisms are not yet fully understood. The composition of gut microbiota plays a crucial role in altering the host immune response, which is closely associated with the development of inflammatory bowel diseases (IBD) [8].

The use of probiotics in UC may help reduce inflammation and improve symptoms, although their effectiveness can vary based on the specific strains used and the severity of the disease [3]. Probiotics can help restore this balance, potentially alleviating symptoms associated with conditions like inflammatory bowel disease. Probiotics exert their effects through immunomodulation, the maintenance of epithelial barrier function, and the modulation of signal transduction pathways. Probiotics can influence the immune system and reduce inflammation through the modulation of T-helper cell responses and the regulation of signalling pathways like TLR4/NF-kB. This immune modulation is essential for managing inflammatory gastrointestinal diseases. These actions can enhance gut health and improve clinical outcomes in patients suffering from gastrointestinal diseases [9,10]. Many COVID-19 patients present with gastrointestinal symptoms like abdominal pain, anorexia, and vomiting [5]. Therefore, many researchers propose that gastrointestinal microbiota homeostasis is changed in COVID-19 patients [6,11]. Severe acute respiratory syndrome coronavirus 2 (SARS-CoV-2) might increase the risk of the morbidity and mortality associated with COVID-19 due to secondary infections in the gastrointestinal tract [12,13].

Probiotics show antiviral effects, and the use of probiotics has been proposed as a cost-effective alternative for managing COVID-19, but research is still emerging. Probiotics are known to enhance the immune response, which may provide some level of protection against viral infections. A recent meta-analysis indicated that probiotics significantly reduced the risk of mortality in COVID-19 patients by 60%. They also decreased the length of hospital stays and recovery times [9]. A systematic review and meta-analysis indicated that probiotics could shorten the duration of symptoms in COVID-19 patients and improve gastrointestinal symptoms. However, while probiotics showed benefits in certain symptoms, no significant improvements were noted for others, such as fever and headache [10].

Other important factors include vitamins, particularly vitamin D, that have been linked to immune regulation and may play a role in managing UC symptoms, although more research is needed to establish definitive benefits [3].

Probiotics may play a role in maintaining balance, potentially impacting the body’s response to respiratory infections like COVID-19. While specific studies on probiotics and COVID-19 are limited, the general benefits of probiotics in supporting immune function suggest they could be beneficial as a complementary approach during viral outbreaks [14]. This study’s aim was to evaluate the possible impact of antibiotic, probiotic, and vitamin use, as well as history of SARS-CoV-2 infection, on the microbiota of UC patients.

## 2. Materials and Methods

### 2.1. Study Design

Forty-nine ulcerative colitis outpatients from Riga East Clinical University Hospital were enrolled in this cross-sectional study from June 2021 to December 2021. Patients older than 18 years old with previously diagnosed UC were included, and all of them signed informed consent. Exclusion criteria were other inflammatory bowel diseases and/or a history of malignant tumours (colon or other). All patients were divided into groups based on history of confirmed SARS-CoV-2 infection (COVID-19 positive vs. COVID-19 negative) in the last 6 months. During an interview, data on vitamin, probiotic, and antibiotic use were collected. Faecal samples were collected from all patients.

Ethical approval for this study was obtained from the Riga East Clinical University Hospital Medical and Biomedical Research Ethical Committee (No. 14/2021). The study adheres to the principles stated in the ‘Declaration of Helsinki’.

### 2.2. Sample Collection and and Metagenomic Analysis

Stool samples were collected in two aliquots by participants at home, using sterile collection tubes without buffer, and within 24 h were delivered to the closest clinical or research laboratory where samples were frozen at −80 °C. Microbial DNA extraction from stool samples was performed using the MGISP-960 Automated Sample Preparation System (MGI Tech Co., Ltd., Wuhan, China) and MagPure Stool DNA LQ Kit (Angen Biotech Co., Ltd., Guangzhou, China). MGIEasy Universal DNA Library Prep Set (MGI Tech Co., Ltd., Wuhan, China) was used for DNA library preparation according to the manufacturer’s instructions, and the following sequencing was done with the DNBSEQ-G400RS sequencing platform using DNBSEQ-G400RS High-throughput Sequencing Set (PE 150) (both from MGI Tech Co., Ltd., Shenzhen, China), providing 150 bp paired-end sequencing reads and obtaining ~30 M reads per sample. For the taxonomical classification of the gut microbiome metagenome data, the MetaPhlAn v.2.6.0 tool was used.

### 2.3. Statistical Analysis

Statistical analyses were performed using R software, version 4.2.1 (R Core Team, 2022, R Foundation for Statistical Computing, Vienna, Austria). Categorical data were summarized as counts (N) and percentages (%). A binomial test was used to evaluate differences between two categorical proportions. Pearson’s chi-square test was applied to analyse independent groups when expected frequencies exceeded five, while Fisher’s exact test was utilized for smaller expected frequencies. Odds ratios (OR) were derived for 2 × 2 contingency tables. The Shapiro-Wilk test assessed whether continuous variables followed a normal distribution. Normally distributed variables were described using the mean (M) and standard deviation (SD), while non-normally distributed variables were expressed as the median (Md) and interquartile range (Q1–Q3). To compare two independent samples, the Student’s *t*-test was employed for normally distributed data; otherwise, the Mann–Whitney U test was used. For comparisons involving three or more groups, analysis of variance (ANOVA) was conducted, with Bonferroni post hoc testing performed if the ANOVA yielded a significant result (*p* < 0.05). Non-parametric comparisons for multiple groups were conducted using the Kruskal–Wallis test. Pearson’s correlation coefficient (r) quantified relationships between normally distributed variables, while Spearman’s rank correlation coefficient (rs) was used for non-normally distributed data. Correlation strength was interpreted as weak (<0.3), moderate (0.3–0.7), or strong (>0.7). Linear regression was employed to examine associations between two quantitative variables, with 95% confidence intervals (CIs) provided to measure parameter precision. Statistical significance was defined as *p* < 0.05 across all tests.

## 3. Results

**Figure 1:**
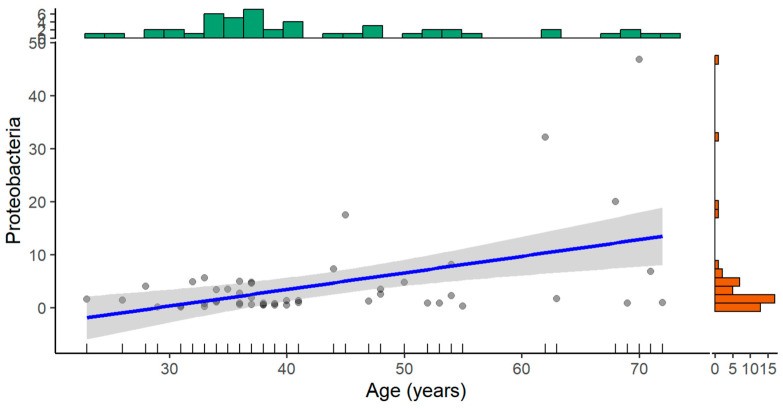
Relationship between Age (years) and Proteobacteria abundance. The blue line represents the fitted regression line showing the trend of Proteobacteria abundance with increasing age. The shaded gray area represents the 95% confidence interval of the regression line. Individual gray dots represent data points for Proteobacteria abundance for each individual. Histograms at the top and right indicate the distribution of Age (years) and Proteobacteria abundance, respectively.

Overall, 49 patients participated in the study. Thirty-one (63%) patients were male and 18 (37%) were female. The median age was 38 years old [IQR: 34; 51]. Based on bacteria phyla analysis, a statistically significant positive correlation between age and amount of *Proteobacteria* was found (rs = 0.47; *p* < 0.01) (Figure 1).

Fourteen patients (28.6%) had a history of SARS-CoV-2 infection. Analysing gut microbiota and COVID-19 status, we found *Verrucomicrobia* to be statistically significantly lower in the COVID-19 positive group (M = 0.05; SD = 0.11) compared to the COVID-19 negative group (M = 0.5; SD = 1.22), *p* = 0.03. (Table 1).

### 3.1. Vitamin Use and Microbiota

**Figure 2:**
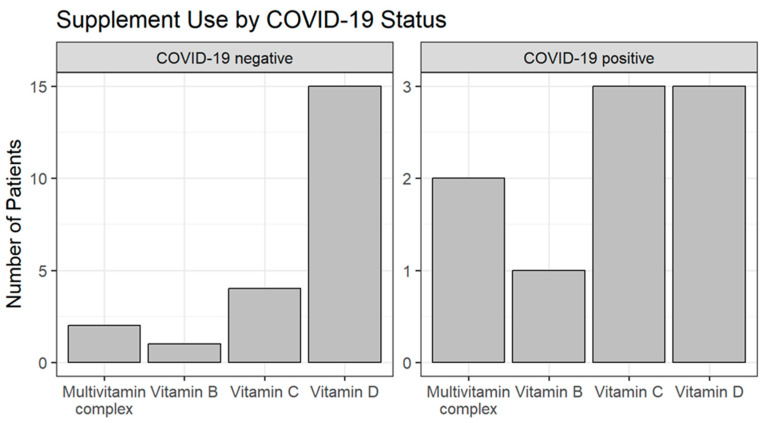
Vitamin supplement use by COVID-19 status.

The most used vitamin supplements were vitamin D and vitamin C. Of the 18 (64.3%) vitamin D users, 3 (21.4%) were COVID-19 positive. Vitamin C was used by 7 (32.8%) patients, of whom 3 (21.4%) were COVID-19 positive, *p* > 0.5. Vitamin B was less frequently used, by only two patients (10%), of whom one was COVID-19 positive (Figure 2).

No statistically significant differences in microbiota composition were found between the patients who used vitamin B and D supplements and those who did not use them (Table 2).

Vitamin C users had a statistically significantly higher abundance of *Firmicutes* in their gut microbiome compared to non-users (Md = 72.8 [IQR: 66.6–78.7] vs. Md = 60.1 [IQR: 42.4–67.7], *p* = 0.01) (Table 3).

### 3.2. Antibiotic Use and Microbiota

**Figure 3:**
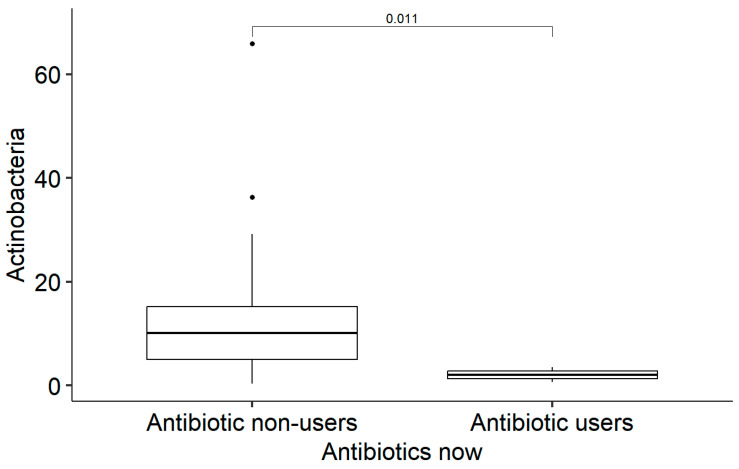
Boxplot showing the distribution of Actinobacteria abundance between antibiotic non-users and antibiotic users. The horizontal line within each box represents the median, and the box indicates the interquartile range (IQR). Whiskers extend to 1.5 times the IQR. Individual dots represent outliers, defined as values that fall outside 1.5 times the IQR above or below the box. The *p*-value (0.011) indicates a significant difference in Actinobacteria abundance between the two groups.

**Figure 4:**
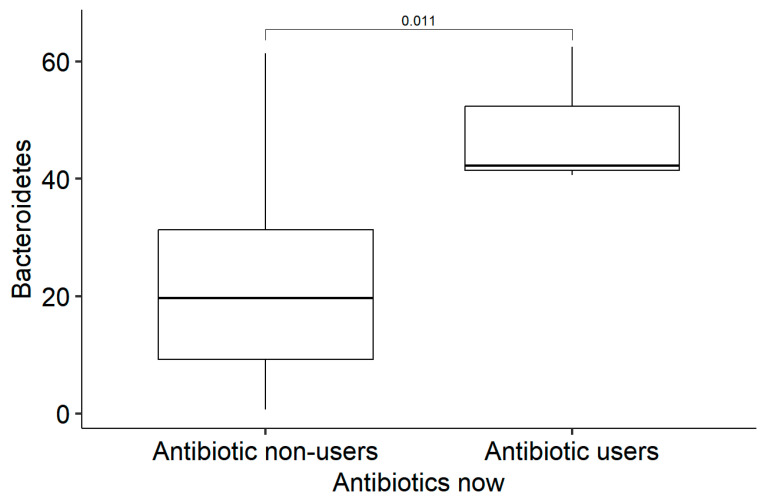
Amount of *Bacteroidetes* in active antibiotic users and non-users.

Only four (11.4%) patients had used antibiotics in the last month before enrolment in the study; of them, three continued their use at the time of faecal sample collection. The prescribed antibiotics were amoxicillin (N = 2), amoxicillin/clavulanic acid (N = 1), and metronidazole (N = 1). Patients who were using antibiotics at the time of faecal sample collection had statistically significantly less *Actinobacteria* in their gut microbiome than non-users (Md = 2.05 [IQR: 1.35; 2.80] vs. Md = 10.1 [IQR: 5.00; 15.2], *p* = 0.01) and more *Bacteroidetes* (Md = 42.2 [IQR: 41.4; 52.3] vs. Md = 19.6 [IQR: 9.27; 31.3], *p* = 0.017) (Figure 3 and Figure 4). No other statistically significant differences in microbiota were found.

Patients who had used antibiotics in the last month had statistically significantly less *Firmicutes* than non-users (Md = 31.5 [IQR: 25.5; 39.8] vs. Md = 64.8 [IQR: 52.3; 69.4], *p* = 0.008), with no other statistically significant differences in microbiota (Table 4).

### 3.3. Probiotic Use and Microbiota

Out of 49 patients, 9 (18.4%) had used probiotics in the last month before enrolment in the study and 3 (6.12%) continued their use of probiotics at the time of faecal sample collection. The most used probiotics were *Saccharomyces boulardii* (N = 4, 15.24%) and a preparation containing *Enterococcus faecium*, *Lactobacillus acidophilus* and *Bifidobacterium infantis* (N = 2, 6.45%). No statistically significant difference in microbiota composition was found between patients who used probiotics recently or were using them at the time of sample collection, and non-users (Table 5).

## 4. Discussion

The comparison of healthy individuals versus those infected with COVID-19 has garnered significant attention in recent years, including the impact of supplementation with vitamin C, D, and probiotics; however, current data remain inadequate to substantiate definitive guidelines regarding the utilization of vitamins or other dietary supplements for the prevention or treatment of COVID-19. Furthermore, it is emphasized that SARS-CoV-2 exerts notable effects on the gut microbiome in both mild and severe cases of COVID-19. Julia S. Galeeva et al. highlights the impacts of SARS-CoV-2 on the gut microbiome among both mild and severe COVID-19 patients without other comorbidities. Differences in alpha and beta diversity in the gut microbiota of mild and severe COVID-19 patients were not statistically significant [15]. However, research comparing patients with different COVID-19 severity levels differs [12,16,17,18]. We did not find statistically significant microbiome differences in COVID-19 positive and negative UC patients.

In patients suffering from ulcerative colitis, the microbiome exhibits distinct alterations when juxtaposed with that of the healthy population. This dysbiosis is believed to stem from a decline in gram-positive Firmicutes, particularly with Clostridium clusters IV and XIVa, alongside an increase in various members of the Proteobacteria phylum, notably Escherichia coli and Enterobacteriaceae. Other bacterial species have also been implicated in UC pathology, including a reduction in the butyrate-producing *Faecalibacterium prausnitzii*, which possesses anti-inflammatory properties, and the mucosa-associated Ruminococcus species, which may contribute to the maintenance of mucosal barrier integrity [19]. A cross-sectional study conducted by Varela et al. posited that the abundance of F. prausnitzii was markedly increased during the remission phase of UC, suggesting its potential role in the therapeutic management of ulcerative colitis [20].

### 4.1. Microbiota and COVID-19

The respiratory and gastrointestinal tracts serve as the primary habitats of the human microbiota and are key targets for SARS-CoV-2 infection [21]. Recent findings indicate significant alterations in the microbiota of COVID-19 patients [22]. A metagenomics analysis conducted on 15 COVID-19 patients hospitalized in Hong Kong revealed a reduction in beneficial commensal bacteria within their faecal microbiomes, accompanied by an increase in opportunistic pathogens. In contrast to the gut microbiomes of healthy persons, the gut microbiomes of COVID-19 patients exhibited diminished levels of bacteria such as Lachnospiraceae, Roseburia, Eubacterium, and *Faecalibacterium prausnitzii*. Notably, Fusicatenibacter showed a markedly reduced prevalence in severe cases and was completely absent in patients who succumbed to the illness [16]. Although there exists a strong correlation between gut bacterial composition and disease severity, the causal relationship between these factors remains ambiguous [16,23].

Metabolites derived from gut microbiota are critical mediators of host–microbiota interactions, significantly influencing host immunity [24]. Among these metabolites are short-chain fatty acids (SCFAs), which are produced by various bacterial groups. SCFAs encompass acetate (50–70%; synthesized by numerous bacterial taxa), propionate (10–20%; generated primarily by Bacteroidetes and certain Firmicutes), and butyrate (10–40%; produced by select Clostridia) [24,25]. SCFAs play a pivotal role in modulating B cell-mediated immune responses in both gut and systemic tissues. Consequently, SCFAs produced by gut microbiota may facilitate anti-SARS-CoV-2 antibody production and mitigate the progression of COVID-19. Research by Zhang et al. indicated that the gut microbiome of COVID-19 patients exhibited impaired SCFA production capabilities, persisting even after the resolution of the disease [26]. Several gut commensals known for their immunomodulatory properties, including *Faecalibacterium prausnitzii*, Eubacterium rectale, and Bifidobacteria, were found to be underrepresented in COVID-19 patients, remaining low post-recovery [13,27]. At the phylum level, Bacteroidetes were found to be relatively more abundant in COVID-19 patients [13,23] compared to non-COVID-19 individuals, whereas Actinobacteria were more prevalent in the latter group [13]. The existing literature suggests that the gut microbiota may remain significantly altered following recovery from COVID-19 [12,13,28]. In our analysis of gut microbiota in patients who had experienced COVID-19 infection within the preceding six months of faecal sample collection, we observed an underrepresentation of Verrucomicrobia in COVID-19 positive patients relative to their COVID-19 negative counterparts. However, conflicting data have emerged from other studies. Dorota Mańkowska-Wierzbicka et al. included subjects with a mild COVID-19 course, revealing a higher abundance of the phylum Verrucomicrobia in COVID-19 patients compared to healthy subjects [15,29,30].

### 4.2. Microbiota and Vitamins

Research indicates that high doses of vitamins, particularly when delivered to the large intestine, can positively influence the gut microbiome. This influence manifests through an increase in the prevalence of beneficial commensal bacteria (vitamins A, B2, D, E, and beta-carotene); the enhancement or preservation of microbial diversity (vitamins A, B2, B3, C, K); the enrichment of microbial richness attributed to vitamin D; increasing short chain fatty acid production (vitamin C); and an increase in SCFA-producing bacteria linked to vitamins B2 and E [31].

Within the human gut microbiome, the dominant bacterial phyla are Firmicutes and Bacteroidetes. The ratio of Firmicutes to Bacteroidetes is thought to play a crucial role in maintaining intestinal homeostasis and overall health, influencing conditions such as obesity, diabetes, and inflammatory bowel disease (IBD) [32]. However, the exact implications of this ratio remain a subject of ongoing debate. Additionally, B vitamins are synthesized by the gut microbiome, and such biosynthesis has been shown to vary according to the host’s health status. Diminished intrinsic production of these vitamins has been observed in individuals with IBD, malnutrition, and metabolic disorders, such as type 2 diabetes mellitus [31,33].

In a study conducted by Harmsen et al. [34], participants who received a high dosage of vitamin B2 (100 mg) over a two-week period exhibited an increase in the count of *Faecalibacterium prausnitzii* per gram of faeces during the supplementation phase, followed by a decrease post-supplementation, although levels did not revert to baseline. *Faecalibacterium prausnitzii* has garnered attention as the primary butyrate producer in the human microbiome, recognised for its anti-inflammatory properties and its role in enhancing gut barrier integrity [31,33]. This study also noted an increase in the *Roseburia* species and a decrease in Escherichia coli, indicating improved anaerobic conditions and redox status within the gut [31]. Furthermore, research by Pham et al. revealed that vitamin B2 supplementation led to an increased diversity of gut species, particularly within the genera Alistipes and Clostridium [31,35].

Vitamin B2 may act as an indirect antioxidant, potentially altering luminal microbiome conditions by reducing luminal reactive oxygen species, thereby fostering a microbiome composition that favours *Faecalibacterium prausnitzii* while diminishing Escherichia coli. The relative increase in *Faecalibacterium prausnitzii* against Escherichia coli could counteract pro-inflammatory mechanisms, suggesting potential therapeutic applications in IBD [31]. However, our study had limitations due to the small number of participants using vitamin B supplements, which precluded the attainment of statistically significant microbiome evaluations.

Vitamin C has demonstrated in vitro antimicrobial properties against a variety of bacterial, fungal, and viral pathogens, suggesting its potential role in modulating intestinal microbial communities [36].

A daily dosage of 500 mg per day for four weeks, as reported by Pham et al., did not yield significant alterations in bacterial composition at the genus or species level. However, there was an observed increase in total SCFA, particularly butyric and propionic acids, following supplementation. No changes in the Bacteroidetes composition were noted with vitamin C treatment [37]. Speculation exists regarding the influence of vitamin C on IBD phenotypes through immune response modulation, yet no correlation was identified between dietary vitamin C and the incidence of ulcerative colitis in participants in the European Prospective Investigation into Cancer and Nutrition (EPIC-Europe) study. Similarly, Buffinton and colleagues reported diminished ascorbate levels in the inflamed mucosa of patients with IBD [31].

In our study, we did not gather data on the dosage of vitamins used, though we found that vitamin C users had statistically significantly more Firmicutes in their gut microbiome. Notably, another study indicated that the combination of vitamin B and vitamin C supplementation resulted in an increase in Firmicutes and a decreased Bacteroidetes [38].

Vitamin D deficiency has been linked to various gastrointestinal tract disorders, including IBD. Recent studies have begun to explore the interactions between vitamin D, its receptor (VDR), the gut microbiome, and inflammation [38,39].

Numerous observational and interventional studies have indicated associations between vitamin D and microbiome composition. Vitamin D supplementation has been shown to induce changes in the faecal microbiome at both the family and species level, including increases in Coriobacteriaceae, decreases in Desulfovibrionaceae, and an elevation of Streptococcus salivarius and Bifidobacterium longum. It is posited that these effects may be partially mediated by the VDR gene, a critical host factor influencing the gut microbiome at the genetic level [35,39]. An open-label pilot study assessing high-dose oral vitamin D supplementation found no significant changes in microbiome composition within the lower gastrointestinal tract or stool samples [40].

A cross-sectional study involving 150 healthy adults examined the relationships between dietary intake of vitamin D3 and circulating 25(OH)D with gut microbiota and inflammatory markers. Participants with the highest vitamin D intake displayed a greater abundance of Prevotella, while Haemophilus and Veillonella were less prevalent [41].

In a randomized, placebo-controlled trial involving 26 overweight or obese, yet otherwise healthy, individuals with vitamin D deficiency, a loading dose of 100,000 IU vitamin D3 followed by 4000 IU daily for 15 weeks resulted in notable changes within the faecal microbiome. The vitamin D-supplemented group exhibited increased levels of Lachnospira and decreased levels of Blautia. Deficient individuals showed a higher abundance of the Clostridiaceae family and Ruminococcus genus, whereas those with sufficient vitamin D levels had an increased proportion of Coprococcus species, especially Coproccous eutactus [42]. Collectively, these studies suggest a modulatory role for vitamin D in the gut microbiome, albeit consistent microbiome alterations across studies remain elusive, with broad trends such as increases in the Bacteroides phylum observed primarily in mouse models [43].

A notable association between vitamin D and IBD has emerged, highlighting a high prevalence of vitamin D deficiency in IBD patients, which correlates with an increased risk of surgery and hospitalization related to IBD [44,45].

Additionally, several studies have investigated the effect of vitamin D supplementation on disease relapse or symptomatology in patients with ulcerative colitis [46,47]. Three mechanisms have been proposed to elucidate the impact of vitamin D on IBD: (1) the modulation of the immune response, particularly through reduced VDR signalling; (2) the enhancement of intestinal epithelial barrier function; and (3) the regulation of gut microbial composition via antimicrobial peptide secretion or direct immune-modulating effects [38].

In our study, we analysed outpatients who previously experienced mild COVID-19 symptoms and did not require hospitalization. We did not observe statistically significant differences in gut microbiota concerning vitamin D usage, nor were there notable differences between the COVID-19 positive and negative groups.

### 4.3. Antibacterial Treatment and Microbiome

In previously mentioned studies, antibiotics were mainly used to treat severe COVID-19 and its complications. As mentioned before, our respondents had mild COVID-19 symptoms and were not hospitalized for this reason. We did not analyse the exact reason for the use of antibacterial treatment. In our study, patients who were using antibiotics at the time of faecal sample collection had statistically significantly less Actinobacteria in their gut microbiome than non-users and more Bacteroidetes than non-users, in comparison with other studies, where Bacteroides decreased after amoxicillin intake and were slower to recover [48,49]. Patients who had been administered antibiotics in the month preceding the collection of faecal samples exhibited a statistically significant reduction in Firmicutes, as corroborated by findings in previous studies [49,50]. Otherwise, there were no statistically significant changes in microbiota between antibiotic users and non-users.

### 4.4. Probiotics and Microbiome

Over the past two decades, numerous publications have investigated the role of probiotics in the management of IBD. One promising approach for the prevention and treatment of IBD involves the modulation of the gut microbiota using probiotics, such as bifidobacteria [51,52]. Probiotics exert their effects against intestinal disorders through a variety of mechanisms, including colonisation, which enhances their therapeutic efficacy. They generate inhibitory compounds such as organic acids, fatty acids, hydrogen peroxide, SCFAs, and bacteriocin-like substances that impede the growth of pathogenic organisms [53].

Supplementation of B. longum and B. breve strains in UC has been linked to reduced disease activity, decreased endoscopic inflammation, and notable anti-inflammatory effects. While bifidobacteria are recognised as key beneficial microorganisms within the human intestinal microbiota and have been extensively studied for their probiotic applications in IBD, the natural prevalence of bifidobacteria within the microbiota of individuals with IBD remains inadequately characterized [54]. We did not find statistically significant differences in gut microbiota depending on probiotic use.

### 4.5. Limitations

In this study, we analysed only ulcerative colitis patients—a population with specific gut microbiota profiles. The study is ongoing, and the patient sample size is increased and includes a control group in further studies and analyses. Additionally, we included patients with history of mild COVID-19 that did not require hospital admission. The quantity, duration, and reasons for supplement use in individuals should be investigated.

## 5. Conclusions

In ulcerative colitis patients, microbiome changes were detected if antibiotics had been used at the time of faecal sample collection or in the last month; less *Firmicutes* and more *Bacteroidetes* were detected. Vitamin C supplement users’ microbiomes had more *Firmicutes* than non-users’. A mild course of coronavirus disease 2019 (COVID-19) may not influence ulcerative colitis patients’ gut microbiome, though *Verrucomicrobia* were underrepresented in COVID-19 positive patients, but more data are needed. The gut microbiome contributes to the pathogenesis of ulcerative colitis, and it is important to understand how vitamins and probiotics can modulate the microbiome and interact with inflammatory bowel disease pathogenesis. Given the high heterogeneity of gut microbiota composition in human populations, we anticipate that a larger sample size will yield more precise and meaningful results.

## Figures and Tables

**Table 1:** COVID-19 status and microbiota composition *.

	COVID-19 Negative, N = 35	COVID-19 Positive, N = 14	OR (95% CI)	*p* Value
Bacteria_Actinobacteria, M (SD)	12.3 (12.2)	10.9 (9.10)	0.99 [0.93; 1.05]	0.67
Bacteria_Bacteroidetes, M (SD)	23.6 (15.5)	23.3 (18.4)	1.00 [0.96; 1.04]	0.94
Bacteria_Firmicutes, M (SD)	57.6 (16.5)	60.9 (20.1)	1.01 [0.97; 1.05]	0.58
Bacteria_Proteobacteria, M (SD)	4.79 (9.55)	3.27 (4.60)	0.97 [0.89; 1.07]	0.46
Bacteria_Verrucomicrobia, M (SD)	0.50 (1.22)	0.05 (0.11)	0.35 [0.04; 3.14]	0.03

* For data analysis, mean values were used, as median is not representative in this case.

**Table 2:** Vitamin D use and microbiota composition.

	Vitamin D Non-Users (N = 31)	Vitamin D Users (N = 18)	*p* Value
Acidobacteria, Md [Q1; Q3]	0.00 [0.00; 0.00]	0.00 [0.00; 0.00]	0.67
Actinobacteria, Md [Q1; Q3]	9.77 [4.02; 15.3]	9.90 [4.79; 12.3]	0.82
Bacteroidetes, Md [Q1; Q3]	21.3 [9.62; 35.2]	18.7 [9.07; 35.0]	0.46
Candidatus Saccharibacteria, Md [Q1; Q3]	0.00 [0.00; 0.00]	0.00 [0.00; 0.00]	0.17
Chlamydiae, Md [Q1; Q3]	0.00 [0.00; 0.00]	0.00 [0.00; 0.00]	0.18
Chlorobi, Md [Q1; Q3]	0.00 [0.00; 0.00]	0.00 [0.00; 0.00]	0.71
Deinococcus Thermus, Md [Q1; Q3]	0.00 [0.00; 0.00]	0.00 [0.00; 0.00]	0.57
Firmicutes, Md [Q1; Q3]	62.0 [48.1; 68.0]	65.4 [53.0; 71.8]	0.43
Proteobacteria, Md [Q1; Q3]	2.19 [0.62; 4.79]	1.12 [0.82; 1.50]	0.26
Verrucomicrobia, Md [Q1; Q3]	0.00 [0.00; 0.00]	0.00 [0.00; 0.11]	0.48

The study included patients with known COVID-19 status (positive or negative within the last six months), but this is not reflected in the grouping of this table.

**Table 3:** Vitamin C use and microbiota composition.

	Vitamin C Non-Users (N = 42)	Vitamin C Users (N = 7)	*p* Value
Acidobacteria, Md [Q1; Q3]	0.00 [0.00; 0.00]	0.00 [0.00; 0.00]	0.56
Actinobacteria, Md [Q1; Q3]	10.9 [4.16; 15.4]	9.43 [5.33; 9.90]	0.23
Bacteroidetes, Md [Q1; Q3]	21.6 [9.55; 35.8]	12.0 [7.68; 27.1]	0.27
Candidatus Saccharibacteria, Md [Q1; Q3]	0.00 [0.00; 0.00]	0.00 [0.00; 0.00]	0.47
Chlamydiae, Md [Q1; Q3]	0.00 [0.00; 0.00]	0.00 [0.00; 0.00]	0.68
Chlorobi, Md [Q1; Q3]	0.00 [0.00; 0.00]	0.00 [0.00; 0.00]	0.56
Deinococcus Thermus, Md [Q1; Q3]	0.00 [0.00; 0.00]	0.00 [0.00; 0.00]	0.4
Firmicutes, Md [Q1; Q3]	60.1 [42.4; 67.7]	72.8 [66.6; 78.7]	0.01
Proteobacteria, Md [Q1; Q3]	1.52 [0.71; 4.73]	1.19 [0.82; 1.37]	0.34
Verrucomicrobia, Md [Q1; Q3]	0.00 [0.00; 0.07]	0.00 [0.00; 0.05]	0.86

The study included patients with known COVID-19 status (positive or negative within the last six months), but this is not reflected in the grouping of this table.

**Table 4:** Antibiotic use during the last month and microbiota composition.

	Did Not Use Antibiotics (N = 45)	Used Antibiotics (N = 4)	*p* Value
Acidobacteria, Md [Q1; Q3]	0.00 [0.00; 0.00]	0.00 [0.00; 0.00]	0.67
Actinobacteria, Md [Q1; Q3]	9.72 [4.08; 13.7]	17.1 [11.6; 30.8]	0.27
Bacteroidetes, Md [Q1; Q3]	19.7 [9.33; 34.6]	34.4 [20.3; 48.2]	0.32
Candidatus Saccharibacteria, Md [Q1; Q3]	0.00 [0.00; 0.00]	0.00 [0.00; 0.00]	0.59
Chlamydiae, Md [Q1; Q3]	0.00 [0.00; 0.00]	0.00 [0.00; 0.00]	0.76
Chlorobi, Md [Q1; Q3]	0.00 [0.00; 0.00]	0.00 [0.00; 0.00]	0.67
Deinococcus Thermus, Md [Q1; Q3]	0.00 [0.00; 0.00]	0.00 [0.00; 0.00]	0.53
Bacteria_Firmicutes, Md [Q1; Q3]	64.8 [52.3; 69.4]	31.5 [25.5; 39.8]	0.008
Proteobacteria, Md [Q1; Q3]	1.32 [0.82; 4.03]	2.77 [0.53; 8.63]	1.000
Verrucomicrobia, Md [Q1; Q3]	0.00 [0.00; 0.08]	0.00 [0.00; 0.09]	0.59

The study included patients with known COVID-19 status (positive or negative within the last six months), but this is not reflected in the grouping of this table.

**Table 5:** Probiotic use in the last month and microbiota composition.

	Probiotic Non-Users (N = 40)	Probiotic Users (N = 9)	*p* Value
Acidobacteria, Md [Q1; Q3]	0.00 [0.00; 0.00]	0.00 [0.00; 0.00]	0.49
Actinobacteria, Md [Q1; Q3]	9.17 [3.71; 15.2]	10.7 [9.43; 12.4]	0.40
Bacteroidetes, Md [Q1; Q3]	20.4 [9.10; 35.8]	23.4 [17.8; 25.5]	0.38
Candidatus Saccharibacteria, Md [Q1; Q3]	0.00 [0.00; 0.00]	0.00 [0.00; 0.00]	0.45
Chlamydiae, Md [Q1; Q3]	0.00 [0.00; 0.00]	0.00 [0.00; 0.00]	0.03
Chlorobi, Md [Q1; Q3]	0.00 [0.00; 0.00]	0.00 [0.00; 0.00]	0.49
Deinococcus Thermus, Md [Q1; Q3]	0.00 [0.00; 0.00]	0.00 [0.00; 0.00]	0.32
Firmicutes, Md [Q1; Q3]	62.2 [49.0; 68.9]	64.6 [50.6; 69.4]	0.97
Proteobacteria, Md [Q1; Q3]	1.48 [0.81; 4.62]	1.19 [0.54; 4.03]	0.47
Verrucomicrobia, Md [Q1; Q3]	0.00 [0.00; 0.06]	0.00 [0.00; 0.12]	0.87

The study included patients with known COVID-19 status (positive or negative within the last six months), but this is not reflected in the grouping of this table.

## Data Availability

The original contributions presented in this study are included in the article. Further inquiries can be directed to the corresponding author.

## Abbreviations

The following abbreviations are used in this manuscript:

CI	confidence interval
COVID-19	coronavirus disease 2019
IBD	inflammatory bowel disease
OR	odds ratio
SARS-CoV-2	severe acute respiratory syndrome coronavirus 2
SCFAs	short-chain fatty acids
UC	ulcerative colitis
VDR	vitamin D receptor

## References

[1] Round J.L., Palm N.W. (2018). Causal effects of the microbiota on immune-mediated diseases. Sci. Immunol..

[2] Wampach L., Heintz-Buschart A., Hogan A., Muller E.E.L., Narayanasamy S., Laczny C.C., Hugerth L.W., Bindl L., Bottu J., Andersson A.F. (2017). Colonization and Succession within the Human Gut Microbiome by Archaea, Bacteria, and Microeukaryotes during the First Year of Life. Front. Microbiol..

[3] Sohail A., Cheema H.A., Mithani M.S., Shahid A., Nawaz A., Hermis A.H., Chinnam S., Nashwan A.J., Cherrez-Ojeda I., Awan R.U. (2023). Probiotics for the prevention and treatment of COVID-19: A rapid systematic review and meta-analysis. Front. Nutr..

[4] Lynch S.V., Pedersen O. (2016). The Human Intestinal Microbiome in Health and Disease. N. Engl. J. Med..

[5] Ansaldo E., Farley T.K., Belkaid Y. (2021). Control of Immunity by the Microbiota. Annu. Rev. Immunol..

[6] Zheng D., Liwinski T., Elinav E. (2020). Interaction between microbiota and immunity in health and disease. Cell Res..

[7] Świrkosz G., Szczygieł A., Logoń K., Wrześniewska M., Gomułka K. (2023). The Role of the Microbiome in the Pathogenesis and Treatment of Ulcerative Colitis—A Literature Review. Biomedicines.

[8] Tian Y., Ran H., Wen X., Fu G., Zhou X., Liu R., Pan T. (2023). Probiotics improve symptoms of patients with COVID-19 through gut-lung axis: A systematic review and meta-analysis. Front. Nutr..

[9] Wang B., Zhang L., Wang Y., Dai T., Qin Z., Zhou F., Zhang L. (2022). Alterations in microbiota of patients with COVID-19: Potential mechanisms and therapeutic interventions. Signal Transduct. Target. Ther..

[10] Sivamaruthi B.S. (2018). A comprehensive review on clinical outcome of probiotic and synbiotic therapy for inflammatory bowel diseases. Asian Pac. J. Trop. Biomed..

[11] Xu R., Lu R., Zhang T., Wu Q., Cai W., Han X., Wan Z., Jin X., Zhang Z., Zhang C. (2021). Temporal association between human upper respiratory and gut bacterial microbiomes during the course of COVID-19 in adults. Commun. Biol..

[12] Zuo T., Zhang F., Lui G.C.Y., Yeoh Y.K., Li A.Y.L., Zhan H., Wan Y., Chung A.C.K., Cheung C.P., Chen N. (2020). Alterations in Gut Microbiota of Patients With COVID-19 During Time of Hospitalization. Gastroenterology.

[13] Yeoh Y.K., Zuo T., Lui G.C.-Y., Zhang F., Liu Q., Li A.Y., Chung A.C., Cheung C.P., Tso E.Y., Fung K.S. (2021). Gut microbiota composition reflects disease severity and dysfunctional immune responses in patients with COVID-19. Gut.

[14] Cox M.J., Loman N., Bogaert D., O’Grady J. (2020). Co-infections: Potentially lethal and unexplored in COVID-19. Lancet Microbe.

[15] Galeeva J.S., Fedorov D.E., Starikova E.V., Manolov A.I., Pavlenko A.V., Selezneva O.V., Klimina K.M., Veselovsky V.A., Morozov M.D., Yanushevich O.O. (2024). Microbial Signatures in COVID-19: Distinguishing Mild and Severe Disease via Gut Microbiota. Biomedicines.

[16] Schult D., Reitmeier S., Koyumdzhieva P., Lahmer T., Middelhoff M., Erber J., Schneider J., Kager J., Frolova M., Horstmann J. (2022). Gut bacterial dysbiosis and instability is associated with the onset of complications and mortality in COVID-19. Gut Microbes.

[17] Xu X., Zhang W., Guo M., Xiao C., Fu Z., Yu S., Jiang L., Wang S., Ling Y., Liu F. (2022). Integrated analysis of gut microbiome and host immune responses in COVID-19. Front. Med..

[18] Righi E., Lambertenghi L., Gorska A., Sciammarella C., Ivaldi F., Mirandola M., Sartor A., Tacconelli E. (2022). Impact of COVID-19 and Antibiotic Treatments on Gut Microbiome: A Role for *Enterococcus* spp.. Biomedicines.

[19] Chehade N.E.H., Ghoneim S., Shah S., Chahine A., Mourad F.H., Francis F.F., Binion D.G., Farraye F.A., Hashash J.G. (2023). Efficacy of Fecal Microbiota Transplantation in the Treatment of Active Ulcerative Colitis: A Systematic Review and Meta-Analysis of Double-Blind Randomized Controlled Trials. Inflamm. Bowel Dis..

[20] Varela E., Manichanh C., Gallart M., Torrejón A., Borruel N., Casellas F., Guarner F., Antolin M. (2013). Colonisation by *Faecalibacterium prausnitzii* and maintenance of clinical remission in patients with ulcerative colitis. Aliment. Pharmacol. Ther..

[21] Lu R., Zhao X., Li J., Niu P., Yang B., Wu H., Wang W., Song H., Huang B., Zhu N. (2020). Genomic characterisation and epidemiology of 2019 novel coronavirus: Implications for virus origins and receptor binding. Lancet.

[22] Zhang H., Ai J.-W., Yang W., Zhou X., He F., Xie S., Zeng W., Li Y., Yu Y., Gou X. (2021). Metatranscriptomic Characterization of Coronavirus Disease 2019 Identified a Host Transcriptional Classifier Associated with Immune Signaling. Clin. Infect. Dis..

[23] Kartsoli S., Vrakas S., Kalomoiris D., Manoloudaki K., Xourgias V. (2022). Ulcerative colitis after SARS-CoV-2 infection. Autops. Case Rep..

[24] Krautkramer K.A., Fan J., Bäckhed F. (2021). Gut microbial metabolites as multi-kingdom intermediates. Nat. Rev. Microbiol..

[25] Dalile B., Van Oudenhove L., Vervliet B., Verbeke K. (2019). The role of short-chain fatty acids in microbiota–gut–brain communication. Nat. Rev. Gastroenterol. Hepatol..

[26] Zhang F., Wan Y., Zuo T., Yeoh Y.K., Liu Q., Zhang L., Zhan H., Lu W., Xu W., Lui G.C. (2022). Prolonged Impairment of Short-Chain Fatty Acid and L-Isoleucine Biosynthesis in Gut Microbiome in Patients With COVID-19. Gastroenterology.

[27] Rocchi G., Giovanetti M., Benedetti F., Borsetti A., Ceccarelli G., Zella D., Altomare A., Ciccozzi M., Guarino M.P.L. (2022). Gut Microbiota and COVID-19: Potential Implications for Disease Severity. Pathogens.

[28] Zuo T., Zhan H., Zhang F., Liu Q., Tso E.Y., Lui G.C., Chen N., Li A., Lu W., Chan F.K. (2020). Alterations in Fecal Fungal Microbiome of Patients With COVID-19 During Time of Hospitalization until Discharge. Gastroenterology.

[29] Ancona G., Alagna L., Alteri C., Palomba E., Tonizzo A., Pastena A., Muscatello A., Gori A., Bandera A. (2023). Gut and airway microbiota dysbiosis and their role in COVID-19 and long-COVID. Front. Immunol..

[30] Mańkowska-Wierzbicka D., Zuraszek J., Wierzbicka A., Gabryel M., Mahadea D., Baturo A., Zakerska-Banaszak O., Slomski R., Skrzypczak-Zielinska M., Dobrowolska A. (2023). Alterations in Gut Microbiota Composition in Patients with COVID-19: A Pilot Study of Whole Hypervariable 16S rRNA Gene Sequencing. Biomedicines.

[31] Pham V.T., Dold S., Rehman A., Bird J.K., Steinert R.E. (2021). Vitamins, the gut microbiome and gastrointestinal health in humans. Nutr. Res..

[32] Khan M.T., Browne W.R., van Dijl J.M., Harmsen H.J.M. (2012). How Can *Faecalibacterium prausnitzii* Employ Riboflavin for Extracellular Electron Transfer?. Antioxid. Redox Signal..

[33] Gehrig J.L., Venkatesh S., Chang H.-W., Hibberd M.C., Kung V.L., Cheng J., Chen R.Y., Subramanian S., Cowardin C.A., Meier M.F. (2019). Effects of microbiota-directed foods in gnotobiotic animals and undernourished children. Science.

[34] Steinert R.E., Sadaghian Sadabad M., Harmsen HJ M., Weber P. (2016). The prebiotic concept and human health: A changing landscape with riboflavin as a novel prebiotic candidate?. Eur. J. Clin. Nutr..

[35] Pham V.T., Fehlbaum S., Seifert N., Richard N., Bruins M.J., Sybesma W., Rehman A., Steinert R.E. (2021). Effects of colon-targeted vitamins on the composition and metabolic activity of the human gut microbiome—A pilot study. Gut Microbes.

[36] Mousavi S., Bereswill S., Heimesaat M.M. (2019). Immunomodulatory and antimicrobial effects of vitamin C. Eur. J. Microbiol. Immunol..

[37] Li L., Krause L., Somerset S. (2017). Associations between micronutrient intakes and gut microbiota in a group of adults with cystic fibrosis. Clin. Nutr..

[38] Shang M., Sun J. (2017). Vitamin D/VDR, Probiotics, and Gastrointestinal Diseases. Curr. Med. Chem..

[39] Chatterjee I., Lu R., Zhang Y., Zhang J., Dai Y., Xia Y., Sun J. (2020). Vitamin D receptor promotes healthy microbial metabolites and microbiome. Sci. Rep..

[40] Bashir M., Prietl B., Tauschmann M., Mautner S.I., Kump P.K., Treiber G., Wurm P., Gorkiewicz G., Högenauer C., Pieber T.R. (2016). Effects of high doses of vitamin D3 on mucosa-associated gut microbiome vary between regions of the human gastrointestinal tract. Eur. J. Nutr..

[41] Luthold R.V., Fernandes G.R., Franco-De-Moraes A.C., Folchetti L.G., Ferreira S.R.G. (2017). Gut microbiota interactions with the immunomodulatory role of vitamin D in normal individuals. Metabolism.

[42] Naderpoor N., Mousa A., Arango L.F.G., Barrett H.L., Nitert M.D., de Courten B. (2019). Effect of Vitamin D Supplementation on Faecal Microbiota: A Randomised Clinical Trial. Nutrients.

[43] Waterhouse M., Hope B., Krause L., Morrison M., Protani M.M., Zakrzewski M., Neale R.E. (2019). Vitamin D and the gut microbiome: A systematic review of in vivo studies. Eur. J. Nutr..

[44] Soltys K., Stuchlikova M., Hlavaty T., Gaalova B., Budis J., Gazdarica J., Krajcovicova A., Zelinkova Z., Szemes T., Kuba D. (2020). Seasonal changes of circulating 25-hydroxyvitamin D correlate with the lower gut microbiome composition in inflammatory bowel disease patients. Sci. Rep..

[45] Ham M., Longhi M.S., Lahiff C., Cheifetz A., Robson S., Moss A.C. (2014). Vitamin D Levels in Adults with Crohn’s Disease Are Responsive to Disease Activity and Treatment. Inflamm. Bowel Dis..

[46] Garg M., Hendy P., Ding J.N., Shaw S., Hold G., Hart A. (2018). The Effect of Vitamin D on Intestinal Inflammation and Faecal Microbiota in Patients with Ulcerative Colitis. J. Crohn’s Colitis.

[47] Garg M., Rosella O., Rosella G., Wu Y., Lubel J.S., Gibson P.R. (2018). Evaluation of a 12-week targeted vitamin D supplementation regimen in patients with active inflammatory bowel disease. Clin. Nutr..

[48] Abeles S.R., Jones M.B., Santiago-Rodriguez T.M., Ly M., Klitgord N., Yooseph S., Nelson K.E., Pride D.T. (2016). Microbial diversity in individuals and their household contacts following typical antibiotic courses. Microbiome.

[49] Elvers K.T., Wilson V.J., Hammond A., Duncan L., Huntley A.L., Hay A.D., van der Werf E.T. (2020). Antibiotic-induced changes in the human gut microbiota for the most commonly prescribed antibiotics in primary care in the UK: A systematic review. BMJ Open.

[50] Khan T.J., Hasan M.N., Azhar E.I., Yasir M. (2019). Association of gut dysbiosis with intestinal metabolites in response to antibiotic treatment. Hum. Microbiome J..

[51] Jakubczyk D., Leszczyńska K., Górska S. (2020). The Effectiveness of Probiotics in the Treatment of Inflammatory Bowel Disease (IBD)—A Critical Review. Nutrients.

[52] Sanders M.E., Merenstein D.J., Reid G., Gibson G.R., Rastall R.A. (2019). Probiotics and prebiotics in intestinal health and disease: From biology to the clinic. Nat. Rev. Gastroenterol. Hepatol..

[53] Jadhav A., Jagtap S., Vyavahare S., Sharbidre A., Kunchiraman B. (2023). Reviewing the potential of probiotics, prebiotics and synbiotics: Advancements in treatment of ulcerative colitis. Front. Cell. Infect. Microbiol..

[54] Bosselaar S., Dhelin L., Dautel E., Titecat M., Duthoy S., Stelmaszczyk M., Delory N., Violante M.D.S., Machuron F., Ait-Abderrahim H. (2024). Taxonomic and phenotypic analysis of bifidobacteria isolated from IBD patients as potential probiotic strains. BMC Microbiol..

